# Parallel use of shake flask and microtiter plate online measuring devices (RAMOS and BioLector) reduces the number of experiments in laboratory-scale stirred tank bioreactors

**DOI:** 10.1186/s13036-015-0005-0

**Published:** 2015-05-30

**Authors:** S. J. Wewetzer, M. Kunze, T. Ladner, B. Luchterhand, S. Roth, N. Rahmen, R. Kloß, A. Costa e Silva, L. Regestein, J. Büchs

**Affiliations:** RWTH Aachen University, AVT - Biochemical Engineering, Worringer Weg 1, 52074 Aachen, Germany; University of Minho, CEB - Centre of Biological Engineering, Campus de Gualtar, 4700-057 Braga, Portugal

**Keywords:** Shake flasks, Microtiter plates, RAMOS, BioLector, RoboLector, Online measurement, Parallel cultivation, Small-scale cultivation, Oxygen limitation, High-throughput

## Abstract

**Background:**

Conventional experiments in small scale are often performed in a ‘Black Box’ fashion, analyzing only the product concentration in the final sample. Online monitoring of relevant process characteristics and parameters such as substrate limitation, product inhibition and oxygen supply is lacking. Therefore, fully equipped laboratory-scale stirred tank bioreactors are hitherto required for detailed studies of new microbial systems. However, they are too spacious, laborious and expensive to be operated in larger number in parallel. Thus, the aim of this study is to present a new experimental approach to obtain dense quantitative process information by parallel use of two small-scale culture systems with online monitoring capabilities: Respiration Activity MOnitoring System (RAMOS) and the BioLector device.

**Results:**

The same ‘mastermix’ (medium plus microorganisms) was distributed to the different small-scale culture systems: 1) RAMOS device; 2) 48-well microtiter plate for BioLector device; and 3) separate shake flasks or microtiter plates for offline sampling. By adjusting the same maximum oxygen transfer capacity (OTR_max_), the results from the RAMOS and BioLector online monitoring systems supplemented each other very well for all studied microbial systems (*E. coli*, *G. oxydans*, *K. lactis*) and culture conditions (oxygen limitation, diauxic growth, auto-induction, buffer effects).

**Conclusions:**

The parallel use of RAMOS and BioLector devices is a suitable and fast approach to gain comprehensive quantitative data about growth and production behavior of the evaluated microorganisms. These acquired data largely reduce the necessary number of experiments in laboratory-scale stirred tank bioreactors for basic process development. Thus, much more quantitative information is obtained in parallel in shorter time.

**Electronic supplementary material:**

The online version of this article (doi:10.1186/s13036-015-0005-0) contains supplementary material, which is available to authorized users.

## Background

**Fig. 1:**
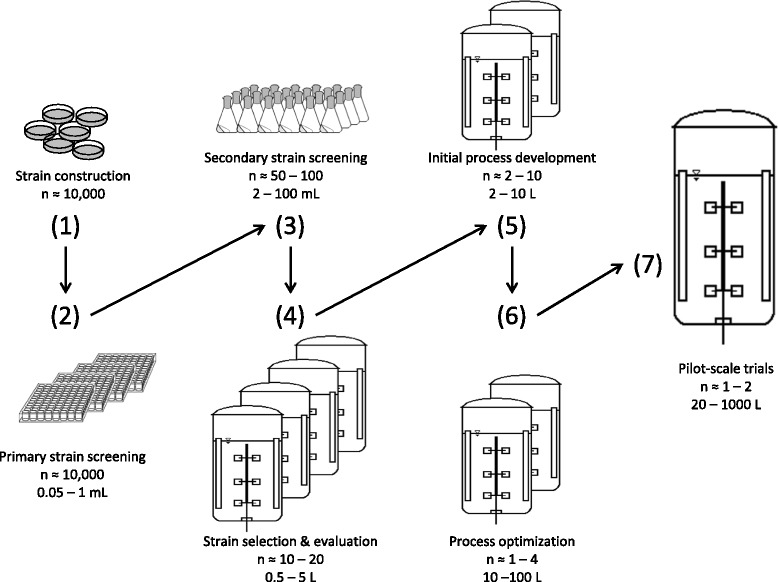
Scheme of an exemplary bioprocess development chain. This scheme represents a conventional development chain of a bioprocess from strain construction to pilot-scale (adopted from Figure one of Bareither and Pollard [1])

In recent years, there has been a constantly increasing demand for high-throughput experimentation in biotechnological applications. Very interesting approaches have been proposed for designing miniature stirred bioreactors with culture volumes as low as 50 μL [1–7]. Yet, microtiter plates (MTP) and shake flasks remain the most commonly used small-scale culture systems that are often applied especially for high-throughput screening [8] and in the early stages of bioprocess development. MTPs are most often used for high-throughput applications, whereas shake flask cultivations are first choice for follow-up experiments. Numerous studies have been conducted to evaluate the performance of a microbial strain under different experimental conditions in order to determine the best suitable candidate for a given process. In both MTP and shake flask systems, however, only little (quantitative) data are gained. Moreover, it is very challenging to perform experiments under controlled fermentation conditions. Therefore, the optimization of process parameters is performed in laboratory-scale stirred tank bioreactors. However, bioreactor size restricts the number of parallel experiments that can be conducted. The sequence, starting at strain construction and ending with pilot-scale trials (compare Fig. 1), results in long development times, termed as a “resource burden” by Bareither and Pollard in 2011 [1]. Furthermore, this whole process may lead to false selection of strains and media due to insufficient data and overlooked unsuitable operating conditions in MTPs and shake flasks. These wrong selections would later adversely affect the whole production process [9]. Consequently, small-scale online monitoring systems such as the Respiration Activity MOnitoring System (RAMOS) and the BioLector devices have been developed [10–16].

With the microtiter plate-based BioLector platform important online information can be optically generated on biomass formation, pH-value and dissolved oxygen tension (DOT) as well as of fluorescence either of respective metabolites (e.g. tryptophan, NADH or riboflavin) or of target proteins eventually tagged by fluorescent marker proteins. Numerous studies have been carried out experimentally proving the value of this high-throughput device [15, 17–20]. However, there is the drawback that the scattered light and the fluorescence signals only provide information relative to a reference and have to be calibrated to obtain quantitative absolute values [21].

As described by Kensy et al., a linear correlation between scattered light and biomass could be obtained to 50 g/L of cell dry weight [18]. Respiration activity available from a stirred tank bioreactor equipped with an exhaust gas analyzer is not provided by the BioLector device. Exactly this missing information can be generated by the RAMOS device [12, 13] on shake flask level. This device measures online the oxygen and carbon dioxide evolution rates in eight parallel shake flasks without the need for calibration. Correct absolute values are provided which can for example also be used to calculate material balances and stoichiometries.

**Fig. 2:**
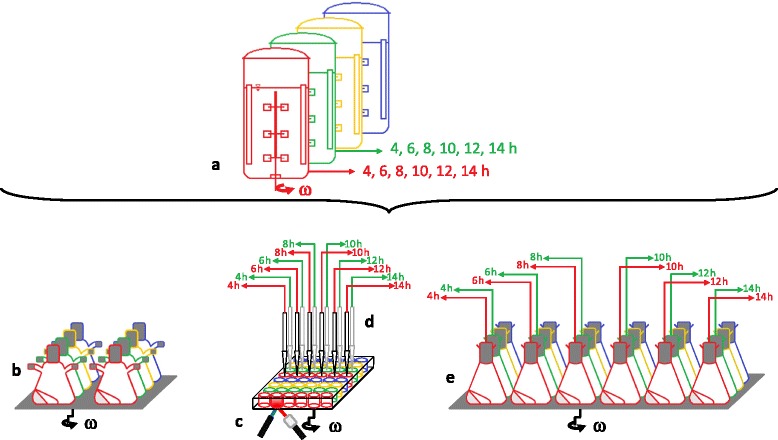
Parallel setup of easy-to-use small-scale culture devices to replace extensive laboratory-scale stirred tank bioreactor experiments. **a** Represents fully equipped laboratory scale stirred tank bioreactors. The culture systems which are commonly used for studying microbial systems (**b**, **c**, **d** or **e**) are operated in parallel. **b** Designates the RAMOS device, (**c**) the BioLector device with (**d**) depicting the RoboLector device (a combination of a BioLector device with an automated liquid handling system), and (**e**) the separate shake flasks for offline sampling. As indicated by different colors in **b**, **c** and **e** four experimental variants can be performed in duplicates in parallel. If only small sample volumes (about 1 mL) are required, the separate shake flasks (**e**) can be replaced by samples automatically taken from a MTP by the RoboLector device (**d**). The specified time points of sampling in (**a**), (**d**) and (**e**) are just examples (i.e. for *E. coli* experiments) and can easily be varied. Only samples taken from the first two rows (encoded in red and green color) are illustrated in (**a**), (**d**) and (**e**) for simplicity

The aim of this current study is to present a new efficient experimental approach (Fig. 2) combining a RAMOS (Fig. 2b) and a BioLector device (Fig. 2c) coupled with an automated liquid handling system (Fig. 2d) or separate shake flasks (Fig. 2e) for offline sampling to reduce the necessary number of experiments in laboratory-scale stirred tank bioreactor (Fig. 2a). Especially we are focusing on step (4) and (5) of a conventional process development chain as depicted in Fig. 1. By coupling the BioLector device with an automated liquid handling system, in whole termed RoboLector (Fig. 2d) [17], automated sampling can be realized, replacing separate shake flasks if only small sample volumes (about 1 mL) are required for analysis. Each separate shake flask or sampling well is withdrawn at specific time points and not reused for later sampling in order to avoid the modification of the well or flask filling volume [22]. Figure 2 presents four experimental variants each coded with a different color. While for each experimental variant one stirred tank bioreactor would be needed, all four variants can be run simultaneously in one RAMOS device in duplicate.

For successful application of the proposed new experimental approach, all bioreactors should be operated at essentially equivalent conditions in order to be considered as one experiment, obtained from one theoretical reactor. Only if this requirement is assured, the microorganisms will behave in an equivalent way. For that goal, the key engineering parameter has to be identified for each microbial system. In this work, the oxygen supply was chosen as the key parameter and, therefore, the maximum oxygen transfer capacity (OTR_max_) in the different bioreactors was kept constant. If the OTR_max_ is constant, then the results from different bioreactors operated in parallel can be combined and regarded as one experiment, similar to the results obtained from one single laboratory-scale stirred tank bioreactor (see Fig. 2). The same starting conditions must be used, because slight differences in the initial optical density (OD_600_) can cause a significant difference in the duration of the lag-phase [17]. Identical starting conditions are achieved by inoculating the total amount of medium (mastermix) required for one experiment. Then, the mastermix is distributed among the different bioreactors resulting in “one experiment”.

Many studies focused on various process-parameters (oxygen transfer, power input, mixing time etc.) as critical scaling parameter and were recently summarized by Marques et al. [23]. Fermentation processes can be successfully scaled-up from shake flasks to laboratory-scale stirred tank bioreactor [24–26] as well as from MTPs to laboratory-scale stirred tank bioreactor [21, 27–29] and even further to pilot-scale stirred tank bioreactor [26, 29]. Scale-up is, therefore, not in the focus of this study. The herein presented approach focusses on the oxygen transfer rate (OTR), which is a key parameter for the evaluation of a cultivation progress. As demonstrated by Anderlei et al. [13], many physiological states of cells - e.g. diauxic growth or limitation by oxygen or by primary as well as secondary substrates - can be identified by the progression of the OTR during a cultivation.

We show that the step of strain selection and strain evaluation as well as the initial process development (step 4 and 5 in Fig. 1) in a laboratory-scale stirred tank bioreactor can be replaced by the new experimental approach (Fig. 2). For the new approach, already existing culture devices can be used. Although for final process optimization experiments (step 6 in Fig. 1) laboratory scale stirred tank bioreactors are still necessary, the total number of stirred tank bioreactor experiments can significantly be reduced.

## Results and discussion

### Determination of the medium-specific maximum oxygen transfer capacity in 48-well microtiter plates and 250 mL shake flasks

To obtain equal cultivation conditions in microtiter plates (48-well Flowerplate) and 250 mL shake flasks, it is important to guarantee equal maximum oxygen transfer capacity (OTR_max_) values. This fact is especially important in case of oxygen limitation which will be examined in a later section.

**Fig. 3:**
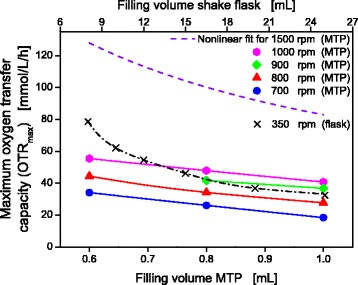
Dependencies of the OTR_max_ for *E. coli* BL21 EcFbFP in shake flasks and 48-well Flowerplate. Culture conditions in RAMOS: WilmsMOPS medium supplemented with 20 g/L glucose, buffered with 0.2 M MOPS, 250 mL RAMOS shake flasks, V_L_ = 8–25 mL, n = 350 rpm, d_0_ = 50 mm, T = 37 °C. Culture conditions for microRAMOS: WilmsMOPS medium supplemented with 20 g/L glucose, buffered with 0.2 M MOPS, 48- well Flowerplate, V_L_ = 0.6 - 1 mL, n = 700–1000 rpm, d_0_ = 3 mm, T = 37 °C. To estimate the OTR_max_ values at a shaking frequency of 1500 rpm, a nonlinear fit, incorporating all data shown for the microtiter plate in this figure, was calculated, thereby resulting in the following equation: OTR_max_ = 1.65 · 10^− 4^ · V_L_^− 0.85^ · n^1.795^

Using *Escherichia coli* BL21 EcFbFP in modified WilmsMOPS mineral medium, OTR_max_ values were determined as described in the Methods section (data shown in Additional file 1: Figure S1, Additional file 2: Figure S2 and Additional file 3: Figure S3). Figure 3 illustrates the dependency of the OTR_max_ value on the filling volume in a 48-well Flowerplate and a 250 mL non-baffled shake flask. For MTP cultivations, different shaking frequencies are displayed, also. In general, a decreasing filling volume results in an increasing OTR_max_ value. Additionally, an increase in the OTR_max_ value can be obtained by increasing the shaking frequency. These findings agree with the results published earlier [8, 12]. The small shaking diameter (3 mm) used for deep well MTP cultivations allows at very high shaking frequencies (n) much higher OTR_max_ and k_L_a values than compared to those of shake flasks which are shaken (maximum at 350 rpm) at a larger shaking diameter (50 mm). As recently reported by Bareither and Pollard, k_L_a values of up to 800 h^−1^ resulting in OTR values of up to 200 mmol/L/h can be reached applying a small shaking diameter of 3 mm paired with a high shaking frequency of 1400 rpm in square-shaped 96-deep-well plates [1]. The herein presented OTR_max_ values for a 48-well Flowerplate (MTP) were obtained by conducting measurements in a MicroRAMOS device as described by Kensy et al. [16] and were restricted to a maximum shaking frequency of 1000 rpm. In order to determine the necessary shaking frequency and filling volume for non-oxygen limited conditions a non-linear regression was made. The regression model incorporates all OTR_max_ values obtained in MTPs for different filling volumes (V_L_) and shaking frequencies (n) ranging from 0.6 mL to 1 mL and 700 rpm to 1000 rpm, respectively. The resulting equation for calculating the OTR_max_ value is:

1 $$ \mathrm{O}\mathrm{T}{\mathrm{R}}_{\max } = 1.65\cdotp {10}^{-4}\cdotp {{\mathrm{V}}_{\mathrm{L}}}^{-0.85}\left[\mathrm{m}\mathrm{L}\right]\cdotp\ {\mathrm{n}}^{1.795}\left[\mathrm{m}\mathrm{i}{\mathrm{n}}^{-1}\right] $$

This equation is specific only for the applied WilmsMOPS mineral medium because a different medium would lead to a different oxygen solubility and different OTR_max_ values. With this equation, OTR_max_ values specific for the cultivation of *E. coli* BL21 EcFbFP in WilmsMOPS mineral medium could be calculated for any combination of shaking frequency and filling volume. As an example, a curve for 1500 rpm and varying filling volumes (0.6 – 1 mL) is shown in Fig. 3.

### Non-oxygen-limited cultivations

Due to the fact that the *E. coli* BSLA EcFbFP culture is still limited applying 8 mL filling volume, 350 rpm shaking frequency and 20 g/L glucose (Additional file 1: Figure S1), there are four factors that can be modified to meet the requirements for non-oxygen-limited condition: (1) decrease the filling volume; (2) increase the shaking frequency; (3) aeration with oxygen enriched air; (4) decrease the sugar concentration. Option 1–3 are not favorable due to practical reasons. A further decrease of the filling volume would lead to unforeseeable evaporation problems. A further increase of the shaking frequency is restricted by the technical design of the orbital shaker. Aeration with oxygen enriched air is expensive, laborious and working with pure oxygen is risky. Therefore, the glucose concentration was decreased and the OTR_max_ values for WilmsMOPS mineral medium supplemented with 10 g/L glucose were determined (see Additional file 3: Figure S3). For 8 mL and 350 rpm no oxygen limitation could be observed. This result indicates that an OTR_max_ value of somewhat more than 70 mmol/L/h has to be guaranteed in order to avoid oxygen limitation in WilmsMOPS mineral medium supplemented with 10 g/L glucose at 37 °C. This prior information about oxygen requirement of the investigated strains is essential and should be generated in primary and secondary strain screening (steps 2 and 3 in Fig. 1).

After the mastermix was prepared, it was distributed among RAMOS flasks (8 mL each), separate shake flasks (8 mL each) and 6 wells of the Flowerplate (1 mL per well). By using one mastermix for all three small-scale culture systems, errors due to medium preparation and inoculation could be avoided. Non-oxygen limited conditions for cultivation with 10 g/L glucose can be expected for shake flask cultivation by applying the following parameters: 50 mm shaking diameter, 350 rpm shaking frequency and 8 mL filling volume resulting in a measured OTR value of 71 mmol/L/h. For the Flowerplate (MTP) the suggested cultivation parameters are: 3 mm shaking diameter, 1500 rpm shaking frequency and 1 mL filling volume resulting in a calculated OTR value of 82.9 mmol/L/h.

**Fig. 4:**
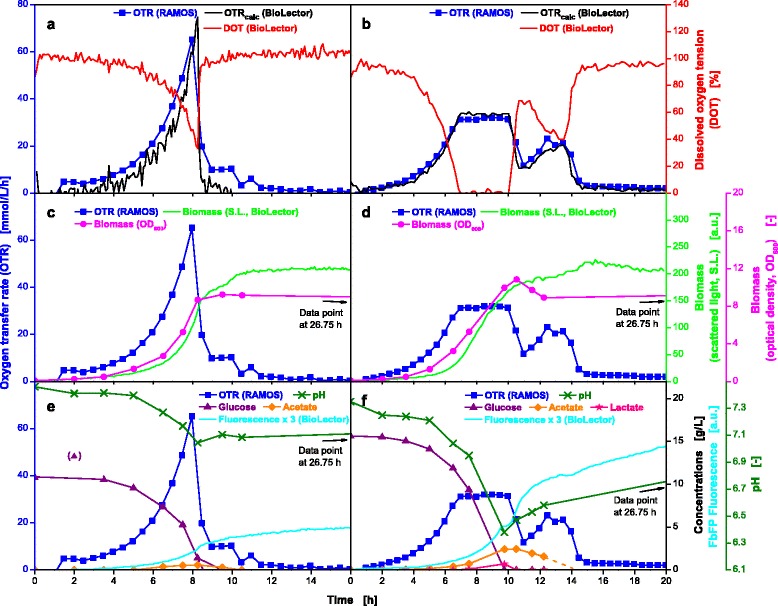
Online and offline data from parallel fermentations of *E. coli* BL21 EcFbFP under non-oxygen-limited and oxygen-limited conditions. Cultivation without oxygen limitation (**a**, **c**, **e**) in WilmsMOPS medium supplemented with 10 g/L glucose, buffered with 0.2 M MOPS. Culture conditions for RAMOS: 250 mL RAMOS shake flasks, V_L_ = 8 mL, n = 350 rpm, d_0_ = 50 mm, T = 37 °C. Culture conditions for BioLector: 48-well Flowerplate, V_L_ = 1 mL, n = 1500 rpm, d_0_ = 3 mm, T = 37 °C. Cultivation under oxygen-limited conditions (**b**, **d**, **f**) in WilmsMOPS medium supplemented with 15 g/L glucose, buffered with 0.2 M MOPS. Culture conditions for RAMOS: 250 mL RAMOS shake flasks, V_L_ = 25 mL, n = 350 rpm, d_0_ = 50 mm, T = 37 °C. Culture conditions for BioLector: 48-well Flowerplate, V_L_ = 1 mL, n = 800 rpm, d_0_ = 3 mm, T = 37 °C. For better visibility, product fluorescence values in (**e**) and (**f**) were multiplied by a factor of three. OTR_calc_ is the calculated OTR course for the MTP determined from the Eq. 2. The k_L_a values for non-oxygen limited and oxygen-limited cultivation were 603 h^−1^ and 183 h^−1^, respectively

Figure 4a, c and e show the results of the non-oxygen-limited cultivation of *E. coli* BL21 EcFbFP. The comparison of the oxygen transfer rate (OTR) and the dissolved oxygen tension (DOT) measured by RAMOS and BioLector devices, respectively, is illustrated in Fig. 4a. The OTR curve demonstrates the characteristic evolution of oxygen consumption over time for a non-oxygen limited *E. coli* cultivation, comparable to previous results [19]. The OTR curve shows no plateau, which indicates that the cultivation was not subject to oxygen limitation at any time. The DOT curve is a mirror image of the OTR curve and displays no oxygen limitation throughout the whole cultivation (DOT ≥ 40 %). Therefore, the estimations for non-oxygen limited conditions made in the previous section were proven to be correct. The OTR curve exponentially increases (DOT curve drops) until complete consumption of the substrate after 8 h. As described in the Methods section, the k_L_a value was calculated by Equation 2 from the DOT allowing the calculation of OTR_calc_ values in the MTPs. As shown in Fig. 4a, the progression of OTR_calc_ in the MTPs and the measured OTR in shake flasks almost concur. However, due to the restriction of the number of measuring points over time for the OTR in shake flasks, the maximum OTR value was not detected. Since the OTR is measured every 30 min and the increase in DOT and the decrease in OTR are identical, most probably the actual OTR peak was missed.

Figure 4c shows that biomass (OD_600_) is produced during the exponential growth phase while the glucose is consumed and small amounts of acetate are produced simultaneously (Fig. 4e) [30]. The end of exponential biomass production is observed at the same time point at which the OTR decreases and the DOT rises (8 h) and was also detected non-invasively, online using the scattered light (S.L., Fig. 4c) measurement installed in the BioLector device. The small increase in the value for S.L. intensity after the OTR curve drops down is most likely due to morphological changes as described in previous publications [31–33].

Between 8 and 10.5 h, the acetate is gradually consumed, leading to the shoulder in the OTR curve. Production and consumption of acetate was also monitored by the pH-value (Fig. 4e). Starting at a value of 7.5 the pH-value steadily decreases to a minimum of 7.0 within 8 h and rises again to a final value of 7.1 as the acetate is consumed. In Fig. 4e, the fluorescence tag signal (EcFbFP) is also plotted as a function over time. The fluorescence activity is relatively low as this culture is not induced by IPTG.

### Oxygen-limited *E.coli* cultivations

Many commonly applied screening conditions lead to oxygen limitation due to unfavorable cultivation parameters. High filling volumes and low shaking frequencies can cause oxygen limitation and, hence, trigger unfavorable effects like formation of products of anaerobic metabolism, excessive pH-shifts and growth inhibition [34, 35]. Characterizing the influence of oxygen limitation is also a relevant subject for scale-up [36], whereas in this case oxygen limitations due to inhomogenities in large scale cannot be compared with the herein triggered oxygen limitations. To simulate oxygen-limited conditions, cultivation parameters were chosen that lead to an OTR_max_ value of about 30 mmol/L/h. For the shake flask cultivation a filling volume of 25 mL at 350 rpm and for the MTP a filling volume of 1 mL at 800 rpm resulted in almost identical OTR_max_ values (Fig. 3).

Figure 4b shows the curves of OTR and DOT for the oxygen-limited cultivation of the same *E. coli* strain discussed in Fig. 4a, c and e. The DOT curve is an inverse of the OTR curve (shake flask) which concurs very well with the OTR_calc_ (MTP). This observation demonstrates that the RAMOS and BioLector devices were operated at conditions providing the same oxygen supply. Combining both measurement techniques, therefore, reveals a great potential. Obviously equivalent OTR_max_ values could successfully be adjusted in shake flasks and MTPs. The exponential growth lasts until 7 h and is then immediately interrupted by the oxygen limitation at an OTR value of 32 mmol/L/h. The plateau of the OTR curve lasts for 3 h while the glucose is consumed before it decreases to 11.8 mmol/L/h and rises again to a maximum of 24 mmol/L/h during consumption of overflow metabolites (Fig. 4f) [37]. It finally decreases to about 0 mmol/L/h after 15 h.

The biomass production under oxygen-limited conditions is displayed in Fig. 4d. The OD_600_ increases exponentially until 7 h followed by a linear progression until 10.5 h. After the maximum OD_600_ of 10.8 is reached, a small drop occurs and afterwards remains constant at a level of about 9. The same trend can also be recognized for online monitored S.L. intensity using the BioLector device (Fig. 4d). The further increase in S.L. after carbon source depletion has already been discussed for non-oxygen limited conditions in the previous section.

Due to the high concentration of overflow metabolites, a diauxic growth can be observed and the OTR shows two peaks (Fig. 4b, d and f) [38]. The formation of 3.1 g/L of total organic acids explains the reduction in the pH-value to 6.4. The last offline sample was taken after 26.75 h and no acetate could be detected. Given the fact that the second OTR peak represents the uptake and consumption of acetate, the dashed line indicates the real progression of the acetate curve and the time point of depletion, which occurs with drop down of the OTR curve after 14 h. For this cultivation, carbon balances have been calculated and could be closed to 96 %. As for non-limited conditions only a low fluorescence signal indicative for product formation is detected due to missing induction.

### Diauxic growth and comparability of offline samples from MTP and shake flask

To challenge our new experimental approach, growth under diauxic conditions was examined using 20 g/L glucose and 1.5 g/L sorbitol as carbon sources [38, 39]. Diauxic growth on several carbon sources results in more distinct peaks and “structure” in the OTR and DOT curves, making comparison of data from shake flasks and microtiter plates more sensitive. As for the previously described experiments, RAMOS, RoboLector and separate shake flasks were run in parallel. In this trial, additional samples for offline analysis were taken directly from the MTP with a RoboLector [17]. Overflow metabolism is intentionally triggered by cultivation conditions that lead to oxygen limitation. Cultivation parameters were chosen using the data from Fig. 3 to obtain equal OTR_max_ values (about 55 mmol/L/h) causing a slight oxygen limitation. Measuring points closest to the desired OTR_max_ value were obtained at 12 mL and 350 rpm in shake flasks and 0.6 mL and 1000 rpm in MTPs. The experiment was performed at 30 °C to slow down the metabolic activity of the *E. coli* strain in order to ensure observability of details of the metabolic behavior. The expected OTR_max_ value will be slightly higher than the value displayed in Fig. 3 because a lower temperature (30 °C instead of 37 °C) results in an increased solubility for oxygen and, therefore, a higher OTR_max_. This is partially compensated by lower diffusion coefficients as shown by Krahe et al. [40].

**Fig. 5:**
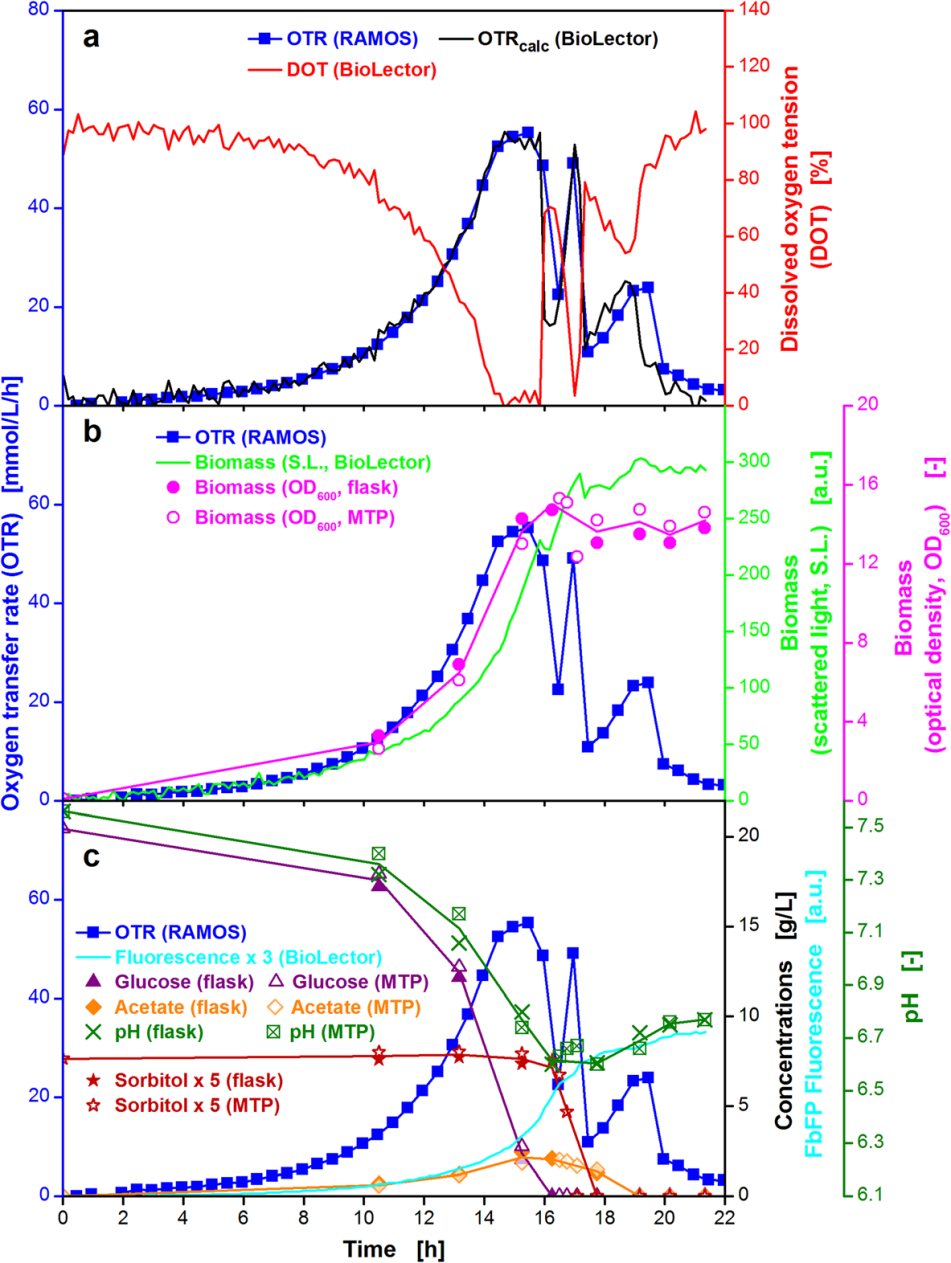
Online and offline data from parallel fermentations of *E. coli* BL21 EcFbFP under diauxic conditions. Cultivation in WilmsMOPS mineral medium supplemented with 20 g/L glucose and 1.5 g/L sorbitol, buffered with 0.2 M MOPS. Culture conditions for RAMOS: 250 mL RAMOS shake flasks, V_L_ = 12 mL, n = 350 rpm, d_0_ = 50 mm, T = 30 °C. Culture conditions for RoboLector: 48-well Flowerplate, V_L_ = 0.6 mL, n = 1000 rpm, d_0_ = 3 mm, T = 30 °C. Lines shown in (**b**) and (**c**) for OD_600_, glucose, sorbitol, acetate and pH represent the averages of the offline data obtained from 250 mL shake flasks and 48-well microtiter plate at the same time points. For better visibility, the sorbitol concentrations were multiplied by a factor of five and the product fluorescence values by a factor of three. The k_L_a value used for calculation of the OTR_calc_ course was (**a**) 276 h^−1^

Figure 5a shows the courses of the OTR and DOT curves of the RAMOS and RoboLector devices, respectively. Additionally, the OTR_calc_ from the DOT values of the MTP is displayed. During the first 17 h, the DOT and OTR_calc_ (MTP) as well as measured OTR (shake flask) match. Due to a slightly higher biomass in the MTP (OD_600_, Fig. 5b) the consumption of overflow metabolites occurs sooner (starting at 17 h) in the MTP cultivation than compared to shake flask cultivation. The OD_600_ values from shake flasks and MTPs were measured, and the results are displayed as circles in Fig. 5b. The line through the circles represents the average of both samples. Even though the cultivation vessels are completely different, the values for OD_600_ agree very well. This indicates that all growth-relevant parameters were identical in shake flasks and MTPs under the applied operating conditions.

Within the first 16 h of incubation, 2.1 g/L acetate is produced and glucose is completely consumed which is indicated by the first drop in the OTR curve also resulting in a decreasing pH-value (Fig. 5c). After this decrease, consumption and depletion of the 1.5 g/L sorbitol takes place within only 1 h (16.5–17.5 h). Subsequently and, in part, simultaneously to the uptake of sorbitol, the pH-value rises because the overflow metabolite acetate is consumed. This observation explains the rise and fall of the OTR curve between 17.5 h and 20 h representing the third peak. Therefore, this culture has to be regarded as a triauxic system [38], although only two different carbon sources have been added at the beginning. Calculating the carbon balance of this microbial system showed that the balance could be closed to a value up to 98 %. Results in Fig. 5b and c demonstrate how OD_600_, the concentration of glucose, sorbitol, acetate and the pH-value concur nicely in the samples from the shake flasks and MTPs. All offline measurements of the different parameters agree very well with each other.

### *E. coli* in mineral auto-induction medium

In a defined mineral auto-induction medium experiment, *E. coli* BL21 BSLA was cultured in parallel in the RAMOS and BioLector devices. The WilmsMOPS mineral auto-induction medium was supplemented with 0.5 g/L glucose as substrate for the initial growth and to repress the *lac* operon [41]. Moreover, the medium contained 2 g/L lactose to induce the *lac* operon (after glucose depletion) and 5 g/L glycerol as additional carbon source. To avoid negative effects of oxygen limitation, conditions were chosen at which the oxygen supply is sufficient in the RAMOS and BioLector devices (OTR_max_ ≥ 60 mmol/L/h).

**Fig. 6:**
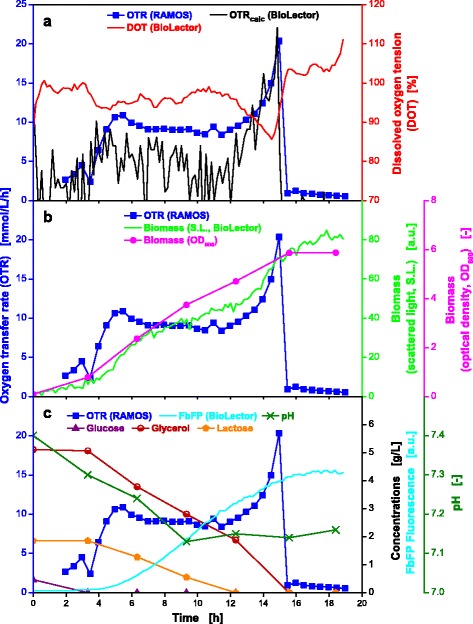
Online and offline data from parallel fermentations of *E. coli* BL21 BSLA in auto-induction medium. Cultivation in WilmsMOPS mineral auto-induction medium containing 0.5 g/L glucose, 2 g/L lactose and 5 g/L glycerol, buffered with 0.2 M MOPS (**a-c**). Culture conditions for RAMOS: 250 mL RAMOS shake flasks, V_L_ = 10 mL, n = 350 rpm, d_0_ = 50 mm, T = 37 °C. Culture conditions for BioLector: 48-well Flowerplate, V_L_ = 1 mL, n = 1500 rpm, d_0_ = 3 mm, T = 37 °C. The k_L_a value used for calculation of the OTR_calc_ course was 587 h^−1^

As shown in Fig. 6a, identically cultivation conditions in the RAMOS and BioLector devices could be established. An initial increase in the OTR curve occurs due to growth on the preferred carbon source glucose. After 3 h, the depletion of glucose (Fig. 6c) leads to a reproducible small drop in the OTR curve. The further increase to a second OTR peak at 10 mmol/L/h after 5 h is due to growth on glycerol. Afterwards, the OTR curve slightly decreases (5–12 h) due to the metabolic burden imposed by the production of the tagged fluorescent protein (FbFP-BLSA) during lactose consumption [42, 43]. After lactose depletion, growth on the residual glycerol leads to an increase in the OTR curve resulting in a third peak at 21 mmol/L/h. The end of the cultivation (after depletion of all carbon sources) is indicated by the sharp drop in the OTR curve. Similar OTR curves were described by Kunze et al. and Rahmen et al. on complex auto-induction medium [44, 45]. The progression of the OTR curve is well mirrored by the progression of the DOT curve. The trend of the calculated OTR in MTPs (OTR_calc_) agrees with the measured OTR curve in shake flasks. However, this example shows the limits of calculating OTR values from DOT values (with the aid of the respective k_L_a values). The accuracy of the OTR directly measured by the RAMOS technique is clearly superior.

The biomass increased exponentially until 6 h of cultivation, followed by a further linear increase (S.L., Fig. 6b). The time point of the shift from exponential to linear growth roughly correlates with the second peak of the OTR curve. The duration of the linear growth (5–12 h) concurs with the expression phase of the fluorescent tagged product (Fig. 6c). With the final increase in the OTR values, additional biomass is produced. The OD_600_ curve points to linear growth throughout the whole cultivation which is most likely attributed to the few sampling points only every two hours. This affirms the value of online monitoring and corresponding signals.

The pH-value changes only moderately, because oxygen limitation and formation of products of anaerobic metabolism could be avoided and, thus, no acidic by-products were formed [37]. The fluorescence signal rises to a value of about 4.5 a.u.

### *Gluconobacter oxydans* cultivations on complex medium

**Fig. 7:**
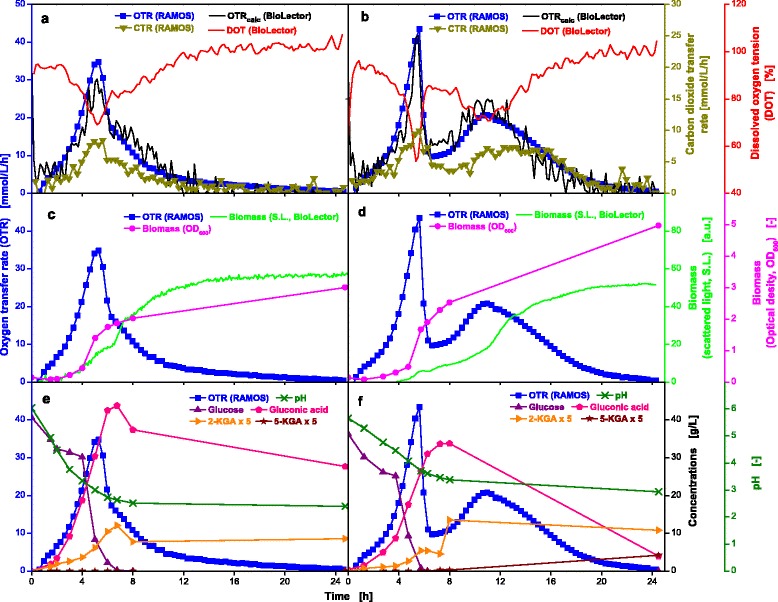
Online and offline data from parallel fermentations of *G. oxydans* 3504 in unbuffered and buffered complex media. Cultivation in unbuffered (**a**, **c**, **e**) and 100 mmol/L PIPPS buffered (**b**, **d**, **f**) complex medium containing 40 g/L glucose. Culture conditions for RAMOS: 250 mL RAMOS shake flasks, V_L_ = 10 mL, n = 350 rpm, d_0_ = 50 mm, T = 30 °C. Culture conditions for BioLector: 48-well Flowerplates, V_L_ = 1 mL, n = 1250 rpm, d_0_ = 3 mm, T = 30 °C. The concentration of the intermediates 2-ketogluconic acid (2-KGA) and 5-ketogluconic acid (5-KGA) were multiplied by a factor of five for better visibility. The k_L_a values used for calculation of the OTR_calc_ course for unbuffered and buffered conditions were 425 h^−1^ and 413 h^−1^, respectively

The acetic acid bacterium *Gluconobacter oxydans* DSM 3504 was additionally tested to verify the proposed new experimental approach for other microorganisms than *E. coli*. Figure 7 displays the comparison of an unbuffered (Fig. 7a, c and xe) and PIPPS buffered (Fig. 7b, d and f) cultivations of the strain *G. oxydans* 3504 under non-oxygen limited conditions. Both experiments were performed simultaneously using a RAMOS and BioLector device. In both cases the OTR curve increases exponentially until it reaches the maximum after 5 h. The first difference between unbuffered and buffered cultivation is visible by the maximum OTR values reached. Under buffered conditions a higher OTR maximum (43.4 mmol/L/h, Fig. 7b) is observed as compared to unbuffered conditions (34.8 mmol/L/h, Fig. 7a). In both cases this behavior is mirror imaged by the DOT value, which drops to a minimum of 69 % and 53 % for the unbuffered and buffered cultivation, respectively. Further on, the OTR steadily decreases for the unbuffered cultivation while for the buffered cultivation a second OTR maximum of 20.8 mmol/L/h is reached after 11 h. Both progressions of the OTR curves are mirrored by the DOT throughout the whole cultivation time. Additionally, the OTR_calc_ courses for MTP cultivations, which were calculated from the measured DOT curves, are in accordance with the measured OTR courses of the shake flasks.

During the cultivation, the CTR is lower than the OTR (RQ < 1), suggesting that not all consumed oxygen was directed into biomass production and that an additional oxidative pathway is active. Taking the curves of the OD_600_ and scattered light measurement (Fig. 7c, d) as well as the progression of the concentration of glucose and its respective oxidation products (gluconic acid, 2-ketogluconic acid and 5-ketogluconic acid, Fig. 7e, f) into account, it becomes clear that the membrane bound oxidases are active, which require a portion of the consumed oxygen [46]. Gluconic acid was continuously formed until glucose was almost depleted and subsequently ketogluconic acids were formed. The formation of 5-ketogluconic acid can only be observed for the PIPPS buffered cultivation because the pH-value remains above 3, allowing glucose dehydrogenase activity [47].

Differences in OD_600_ and S.L. become obvious in Fig. 7c, d. While for unbuffered conditions the OD_600_ and S.L. curves partly concur, they show different courses for the buffered cultivation. This observation may be caused by an altered morphology of the cells as described by Kunze et al. [48]. As recently published, soluble buffers significantly increase the osmotic stress and therefore influence the oxygen consumption of *G. oxydans* 3504 [49]. Hence, it is highly conceivable that the osmotic pressure also influences the cell morphology.

Two different experiments (unbuffered and 100 mmol/L PIPPS) were simultaneously preformed resulting in a data density acquired so far only from cultivations in fully equipped laboratory-scale stirred tank bioreactors. Additionally, very interesting findings could be made by monitoring the S.L. intensity and OD_600_ values during the cultivations. Two additional cultivations were performed simultaneously as well, with different buffer concentrations (data not shown) which adds up to four different experimental variants at the same time as illustrated in Fig. 2. As an important advantage, all four experimental variants were performed in duplicate (in RAMOS) and in triplicate (in BioLector) to verify the repeatability of the obtained results.

### Oxygen-limited cultivation of the yeast *Kluyveromyces lactis* on complex medium

In order to prove the applicability of the proposed new experimental approach for yeast cultivations, the genetically modified strain *Kluyveromyces lactis* Cel5A was investigated. In addition to oxygen limitation, a high substrate concentration (30 g/L galactose) was applied to cause overflow metabolism.

**Fig. 8:**
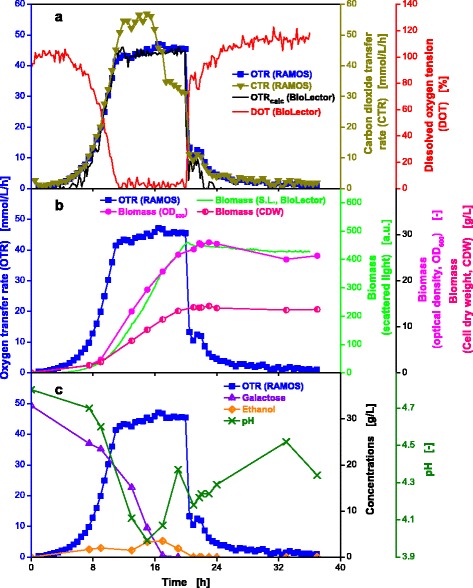
Online and offline data from parallel fermentations of *K. lactis* Cel5A. Growth under oxygen-limited conditions in complex YEP-medium supplemented with 30 g/L galactose (**a-c**). Culture conditions for RAMOS: 250 mL RAMOS shake flasks, V_L_ = 20 mL, n = 350 rpm, d_0_ = 50 mm, T = 30 °C. Culture conditions for BioLector: 48-well Flowerplates, V_L_ = 1 mL, n = 800 rpm, d_0_ = 3 mm, T = 30 °C. The k_L_a value used for calculation of the OTR_calc_ course was 203 h^−1^

The curves for OTR and DOT plotted in Fig. 8a show a steep increase or decrease, respectively, which is caused by an exponential growth phase until 11 h. During 12 and 20 h, the yeast grows linearly due to the oxygen-limited culture conditions. Both signals, OTR and DOT, from shake flask and microtiter plate are in agreement with respect to start and end of oxygen limitation. The OTR_calc_ calculated from the DOT values of the MTP (Eq. 2) concurs nicely with the measured OTR in shake flasks, suggesting equivalent environmental conditions for the microorganism in shake flasks and MTPs. The hypothesis of changing from exponential to linear growth can be verified by the evolution of the biomass curves (S.L. intensity, OD_600_ and cell dry weight) plotted in Fig. 8b. All three biomass values (online and offline) show the same course, which indicates that an influence of the cell morphology on the scattered light intensity for *K. lactis* can be excluded under these specific conditions. The offline data presented in Fig. 8c, show that galactose is used as carbon source and ethanol is produced simultaneously as previously described in literature [50]. The depletion of galactose is barely indicated by the OTR curve, whereby only a short small increase at 17 h is visible (Fig. 8a). However, the CTR curve indicates a drastic change in respiration behavior. After depletion of galactose and initiation of ethanol consumption, the CTR level drops from about 55 to 35 mmol/L/h, while the OTR level remains constant at 45 mmol/L/h. Depletion of ethanol causes a simultaneously drop in the OTR and CTR curve and the DOT value rises. Between 21 and 22 h, the OTR and CTR curves show another shoulder which is mirrored by the DOT as well (Fig. 8a). Given the fact that no further secondary metabolites were detected in HPLC analysis, the cells supposedly grow on complex components of the YEP medium. Nevertheless, the carbon balance was closed to 99 %.

The decrease in the pH-value during the first 16 h is attributed to the uptake of ammonium salts from the medium as stated in literature [51]. The subsequent rise and fall of the pH-value can be explained by the uptake of complex proteinaceous components from the medium and the release of ammonium. Because the use of complex media makes a mechanistic interpretation of results much more difficult than results obtained with a defined mineral medium, one should avoid complex media. Complex media components resulting in high turbidity of the medium cause obvious problems when working with optical measurements.

## Conclusions

This study has investigated the feasibility of a new experimental approach operating two culture- and online-monitoring devices based on shake flasks and microtiter plates (RAMOS and BioLector) in parallel to acquire comprehensive data on growth and production behavior of microorganisms. The obtained results are very helpful to understand complex metabolic relationships under different cultivation conditions such as diauxic growth, oxygen limitation and auto-induced product formation.

The operating conditions in shake flasks and microtiter plates can be precisely adjusted (for equivalent OTR_max_) so that the microorganisms behave in an equivalent way over time. This conclusion is supported by the observation that the OTR in shake flasks and DOT signals in microtiter plates are mirror images of each other and the OTR calculated from the DOT measured in MTPs concurs with the measured OTR in in shake flasks. Additionally, the comparison of offline data from the MTP and shake flasks shows identical values demonstrating that equal conditions were applied during the cultivation. That the new approach is functional and effective could also be shown by the closed carbon balances. The preparation of one common mastermix for one experiment, before the inoculated medium is distributed among the different bioreactors, is an essential component of the new approach.

With this experimental technique problems like different lag-phases in the parallel running small-scale cultivation systems can be avoided and, therefore, we do not need to rely on calculating rates in the different systems. As four or even eight different experiments can be performed in parallel under very well controlled conditions, providing all the information usually obtained from laboratory-scale stirred tank bioreactors (equipped with an exhaust gas analyzer) the proposed experimental approach is very time and cost efficient. Therefore, the number of extensive experiments in laboratory-scale stirred tank bioreactors, related to strain selection and evaluation during initial process development (step 4 and 5 in Fig. 1), can be reduced by the suggested combination of small-scale culture systems.

The described approach is currently limited to batch experiments using pH buffers. However, it is imaginable to extend this approach also to fed-batch cultures, applying polymer based controlled release systems [52–55], enzymatic release systems (restricted to glucose) [56–62] or diffusion controlled feeding of compounds [63, 64]. With the first and last mentioned techniques also cultures with some degree of pH control at strongly reduced buffer concentration can be performed [64]. Additionally Funke et al. described a disposable and user-friendly microfluidic system for MTPs, which allows pH-controlled batch cultivations as well as fed-batch cultivations and show very good comparability with a laboratory-scale stirred tank bioreactor [28]. Investigations are currently performed to combine the aforementioned techniques for shake flasks and the herein presented new experimental approach, to make pH-controlled and fed-batch cultivations feasible.

## Methods

### Materials

Chemicals were obtained from Sigma-Aldrich (Taufkirchen, Germany), Qiagen (Hilden, Germany), Merck (Darmstadt, Germany), Roth (Karlsruhe, Germany), and Roche Diagnostics (Mannheim, Germany).

### Strains

In this study, four different strains were investigated: three bacterial strains, i.e., *Escherichia coli* BL21 (DE3) pRhotHi-2-EcFbFP-His6 (*E. coli* BL21 EcFbFP), *E. coli* BL21 (DE3) pET22b(+)-His6-FbFP-BSLA-Ile12Cys (*E. coli* BL21 FbFP BSLA) and *Gluconobacter oxydans* DSM 3504, as well as a yeast strain, i.e., *Kluyveromyces lactis* Cel5A.

#### Bacterial strains

As cited above, two slightly different *E. coli* BL21 (DE3) strains were used. The first one expresses a FMN-based fluorescence protein (FbFP), codon-optimized for *E. coli* as expression host (hence, termed EcFbFP) [65, 66]. This strain was kindly provided by Dr. Drepper from the group of Prof. Jaeger from the Institute for Molecular Enzyme Technology, Heinrich Heine University Düsseldorf.

The second strain, *E. coli* BL21 FbFP BSLA expresses *Bacillus subtilis* lipase A (BSLA) fused to FbFP and exhibits an amino acid exchange from isoleucine to cysteine at position 12 [65, 67]. This strain was also kindly provided by the group of Prof. Jaeger.

*Gluconobacter oxydans* DSM 3504 wild-type was kindly provided from the group of Prof. Liebl from the Technical University Munich, Department for Microbiology.

#### Yeast strain

*Kluyveromyces lactis* Cel5A was derived from the parental strain *K. lactis* GG799 which is a haploidic wild-type strain used in the food industry. The gene for the endocluconase Cel5A (EG III; UniProt-Nr.: P07982) was derived from *Trichoderma reesei* and cloned into *K. lactis* GG799 using the integrative vector pKLAC1. Data for product formation is not shown because it is not focus of this work. The strain was kindly provided from the group of Dr. Commandeur from RWTH Aachen, Institute for Molecular Biotechnology.

### Media

#### *E. coli* precultivation media

To promote growth of *E. coli*, two precultivations with different media were performed. The first preculture was carried out in complex Terrific Broth (TB) [68] with the medium composed of 12 g/L tryptone, 24 g/L yeast extract, 12.54 g/L K_2_HPO_4_, 2.3 g/L KH_2_PO_4_ and 5 g/L glycerol dissolved in deionized water. The pH-value was 7.2 ± 0.2 without adjustment. Modified WilmsMOPS mineral medium was used for the second preculture and was prepared according to Wilms et al. [69]. The mineral medium consisted of 5 g/L glycerol, 5 g/L (NH_4_)_2_SO_4_, 0.5 g/L NH_4_Cl, 3 g/L K_2_HPO_4_, 2 g/L Na_2_SO_4_, 0.2 M (N-Morpholino)-propanesulfonic acid (MOPS), 0.5 g/L MgSO_4_ · 7H_2_O, 0.01 g/L thiamine hydrochloride, 1 mL/L trace element solution [0.54 g/L ZnSO_4_ · 7H_2_O, 0.48 g/L CuSO_4_ · 5H_2_O, 0.3 g/L MnSO_4_ · H_2_O, 0.54 g/L CoCl_2_ · 6H_2_O, 41.76 g/L FeCl_3_ · 6H_2_O, 1.98 g/L CaCl_2_ · 2H_2_O, 33.4 g/L Na_2_EDTA (Titriplex III)]. The pH-value was adjusted to 7.5 using 1 M NaOH. All medium components were sterilized separately by autoclaving or filtration before mixing. The concentration and type of C-source is specified in the figure legends.

### *E. coli* main culture media

#### Non-inducing growth conditions in mineral medium

For non-inducing growth conditions (non-oxygen-limited and-limited), the same modified WilmsMOPS mineral medium was used as described for the second precultivation in the section above. No glycerol was added; the respective concentrations of sugars are specified in the figure legends.

#### Inducing growth condition in mineral auto-induction medium

If protein production ought to be induced, the previously described modified WilmsMOPS mineral medium for the second precultivation is supplemented with 0.5 g/L glucose for initial growth and 2 g/L sterilized lactose as inducing compound [70–72]. The glycerol concentration is kept at 5 g/L.

#### Diauxic growth

To examine diauxic growth behavior, modified WilmsMOPS mineral medium was prepared as mentioned for the second preculture but with altered substrate concentrations. No glycerol was added to the medium but rather a total of 20 g/L glucose and 1.5 g/L sorbitol.

### *Gluconobacter* medium

The complex culture medium for *G. oxydans* DSM 3504 was prepared as described by Richardt et al. 2012 and was composed of 5 g/L yeast extract, 1 g/L KH_2_PO_4_, 1 g/L (NH_4_)_2_SO_4_ and 2.5 g/L MgSO_4_ 7H_2_O [73]. The pH was adjusted to 6.0 with 1 M KOH. After sterilization at 121 °C for 21 min, concentrated separately autoclaved glucose solution was added to obtain a final concentration of 40 g/L glucose. Where indicated, the medium was supplemented with concentrated PIPPS-buffer solution to a final concentration of 100 mmol/L.

### *Kluyveromyces* medium

For the cultivation of *K. lactis* Cel5A, a basic complex YEP-medium was used. This consisted of 10 g/L yeast extract and 20 g/L peptone. The pH-value was adjusted to 4.8 using 1 M HCl prior to sterilization at 121 °C for 21 min. After the medium was allowed to cool down, concentrated separately autoclaved galactose solution was added to obtain a final concentration of 30 g/L galactose.

### Cultivation parameters

For all main cultivations, the so called ‘mastermix’ (medium with inoculated microrganisms) was prepared as follows: The medium for the main cultivation was made by adding the required supplements, and the beforehand determined volume of preculture to obtain the desired initial optical density.

#### *E. coli* precultivations

Precultivations of *E. coli* BL21 EcFbFP were performed in regular 250 mL non-baffled shake flasks. For the first preculture, 10 mL TB-medium containing 50 μg/mL kanamycin was inoculated with 100 μL from a cryo stock (cell solution in 50 % (w/w) glycerol/water). Subsequently the non-baffled shake flasks were incubated overnight at a temperature (T) of 37 °C on an orbital shaker at a shaking frequency (n) of 350 rpm and a shaking diameter (d_0_) of 50 mm. As soon as an OD_600_ of 17 ± 3 was reached (6–18 h), the second preculture was started by inoculating 10 mL of modified WilmsMOPS mineral medium supplemented with 10 g/L glucose to an initial OD_600_ of 0.1. Cultivation parameters (T, n, d_0_) were maintained as mentioned above, and the culture was incubated overnight for 8–11 h until an OD_600_ of 9 ± 2 was obtained.

Precultivations for *E. coli* BL21 BSLA were carried out in modified 250 mL non-baffled shake flasks in an in-house RAMOS device [13, 12] by applying a filling volume of 10 mL. For incubation at T = 37 °C, an orbital shaker was used with the shaking parameters n = 350 rpm and d_0_ = 50 mm. For the first precultivation, 10 mL of TB medium containing 100 μg/mL ampicillin were inoculated with 100 μL from a cryo stock (cell solution in 50 % (w/w) glycerol/water), incubated for 3 h and harvested as soon as an OTR value of 40–50 mmol/L/h had been reached. An aliquot of 10 mL of modified WilmsMOPS mineral medium supplemented with additional 0.5 g/L glucose was inoculated with culture broth from the first precultivation to start the second precultivation. To achieve an initial optical density at 600 nm (OD_600_) of 0.1, the respective volume of culture broth from the first precultivation was added. Cultures were grown under the same incubation parameters (T, n, d_0_) for 5 h and harvested at an OTR of 25–35 mmol/L/h.

#### *E. coli* main cultivations

Processing of the main cultures for both *E. coli* strains were identical. For all cultivations, a mastermix with an initial OD_600_ of 0.1 was prepared. Subsequently, the mastermix was divided among to the RAMOS flasks, microtiter plates and separate shake flasks (cultivation parameters are described in a later section). The respective volumes are specified in the figure legends.

#### Cultivation of *Gluconobacter oxydans*

Precultivations of *G. oxydans* were performed in 10 mL of the aforementioned complex medium in 250 mL non-baffled shake flasks, without the addition of glucose. An antibiotic, 50 μg/mL cefoxitin sodium, was added prior to inoculation with 1 mL from a cryo stock (cell solution in 50 % (w/w) glycerol/water). The incubation was conducted overnight (12–14 h) on an orbital shaker at 30 °C with n = 350 rpm and d_0_ = 50 mm. After determination of the OD_600_, the required volume of cell broth to inoculate the main culture to an initial OD_600_ of 0.1 was calculated and transferred to a centrifugation tube to separate cells and medium. The harvested bacterial cells were re-suspended in fresh culture medium and subsequently transferred to the mastermix and distributed among RAMOS flasks, MTPs and separate shake flasks.

#### Cultivation of *Kluyveromyces lactis*

As preculture, an aliquot of 10 mL of YEP medium (without galactose) was filled into a 250 mL non-baffled shake flask and was inoculated with 100 μL from a cryo stock (cell solution in 50 % (w/w) glycerol/water). After incubation at 30 °C with n = 350 rpm and d_0_ = 50 mm for 12–14 h the cells were harvested at an OD_600_ value of 20 ± 3. Subsequently, the desired amount of culture volume was centrifuged, the supernatant was discarded, and the cell pellet was re-suspended in fresh YEP medium to an initial OD_600_ of 0.1.

#### Shake flask cultivations in the RAMOS device

The in-house developed Respiration Activity MOnitoring System (RAMOS) [12, 13] allows online measurement of the oxygen transfer rate (OTR), carbon dioxide transfer rate (CTR) and of the respiratory quotient (RQ). Commercial versions of the RAMOS device are available from Kuhner AG, Birsfelden, Switzerland or HiTec Zang GmbH, Herzogenrath, Germany. The resulting characteristic curves provide information about growth and metabolic activity of the investigated microorganism. All cultivations were conducted with the individually specified culture conditions in 250 mL non-baffled shake flasks which were modified as described by Anderlei and Büchs [13].

#### Microtiter plate cultivations in the BioLector device

All microtiter plate (MTP) cultivations were performed in 48-well Flowerplates (m2p-labs, Baesweiler, Germany). For sterile cultivation conditions the plates were cover with an adhesive gas-permeable membrane (Thermo Scientific, Dreieich, Germany). An in-house developed BioLector device was used for non-invasive online measurement of scattered light (signal for biomass formation) and fluorescence (signal for product formation) during cultivations [15, 18]. Commercial versions of the BioLector device are available from m2p-labs GmbH, Baesweiler, Germany. Signals were obtained by irradiating every single well with light of a defined wavelength (excitation), and detecting and analyzing the reflected/scattered light or fluorescence. In combination with special microtiter plates equipped with optodes on the bottom of the MTPs, dissolved oxygen tension (DOT) can be measured fluorometrically quasi-online and non-invasive. If not otherwise stated all BioLector cultivations were monitored with the adjustments summarized in the Table 1.

**Table 1:** Overview of excitation and emission wavelengths in the BioLector

Signal	Filter (excitation/emission)	Gain
*E.coli* BL21 EcFbFP		
Scattered light	620 nm/-	30
FbFP-fluorescence	450 nm/492 nm	60
pO_2_-optode	520 nm/600 nm	60
pH -optode	470 nm/525 nm	45
*E.coli* BL21 BSLA		
Scattered light	620 nm/-	30
FbFP-fluorescence	450 nm/492 nm	60
pO_2_-optode	520 nm/600 nm	60
*G. oxydans* 3504		
Scattered light	620 nm/-	30
pO_2_-optode	520 nm/600 nm	50
*K. lactis* Cel5A		
Scattered light	620 nm/-	30
pO_2_-optode	505 nm/590 nm	50

#### RoboLector system

The BioLector device can be combined with various automated liquid handling systems (“pipetting robots”), which allows for experiment automation, including automated sampling [17]. Meanwhile this and comparable concepts have also been adapted by other research groups [56, 74, 75].

#### Shake flask cultivations

Shake flask cultivations were carried out in conventional 250 mL non-baffled Erlenmeyer flasks with the respective filling volume (specified in figure legends) under identical cultivation conditions as described for the main cultivations. At different time points, single flasks were removed from the shaker and completely used for sampling. Carbon source and by-product concentrations were determined by HPLC. Moreover, OD_600_, cell dry weight and the pH-value were measured offline.

#### OTR_max_ determination

For determination of the maximum oxygen transfer capacity (OTR_max_) in shake flasks, the main culture of *E. coli* BL21 EcFbFP was grown on modified WilmsMOPS mineral medium containing 20 g/L glucose and no glycerol. While keeping the shaking diameter (50 mm) and the shaking frequency (350 rpm) constant, the filling volume was altered in a range of 8–25 mL. The plateau in the resulting curves equals the OTR_max_ value under the respective conditions (see Additional file 1: Figure S1).

In order to determine the maximum oxygen transfer capacity of a Flowerplate a modified RAMOS device was used which was first described 2005 by Kensy et al. [16]. The so-called MicroRAMOS is capable of measuring the oxygen transfer rate of a whole microtiter plate. The cultures were grown in WilmsMOPS mineral medium supplemented with 20 g/L glucose and no glycerol. A high initial optical density (OD_600_) of 1.0 was established to shorten the lag phase, thereby enabling the formation of multiple plateaus within one experimental run. For determining OTR_max_, the shaking diameter was kept constant at 3 mm whereas the shaking frequency and the filling volume were altered in a range of 500–1000 rpm and 0.6–1.0 mL, respectively. The attained plateaus represent the OTR_max_ value of the applied cultivation condition (see Additional file 2: Figure S2).

### Software

All calculations were performed with MATLAB (Version R2014a 8.3.0.532, The MathWorks, Inc., Natik, USA).

#### Calculation of the oxygen solubility

The oxygen solubilities for the respective cultivation media and temperatures were estimated according to Wilhelm et al. [76], Weisenberger & Schumpe [77] and Rischbieter & Schumpe [78]. Since the influence of Good’s buffers was not investigated in these studies a hi-value of 0.2 m^3^/kmol for MOPS and PIPPS was assumed with respect to the molecular structure of investigated substances. Furthermore, the influence of sorbitol and galactose on the oxygen solubility was regarded as equal to the influence of glucose. Changes of the oxygen solubility during the cultivation due to changes of the media composition were not taken into account.

#### Approximation of k_L_a values based on measured DOT and OTR

Since the time vectors of the OTR measurements in the shake flask system (measurement point every 0.5 h) and DOT measurements in the MTP (measurement point every 0.09 h) were not identical, both time vectors were combined. Missing OTR and DOT values were interpolated with a cubic spline (MATLAB function: interp1, default settings). For each time point of the combined time vector corresponding values for OTR and DOT were available with these interpolated values. The oxygen transfer rate (OTR_calc_) in MTPs is calculated from the measured DOTs according to the following equation:

2 $$ \mathrm{O}\mathrm{T}{\mathrm{R}}_{\mathrm{cal}\mathrm{c}} = {\mathrm{k}}_{\mathrm{L}}\mathrm{a}\ \cdotp\ {\mathrm{L}}_{\mathrm{O}2}\cdotp\ \left(\mathrm{p}{{\mathrm{O}}_2}^{\mathrm{gas}}-\left(\mathrm{DOT}/100\right)\kern0.22em \cdotp \kern0.22em \mathrm{p}{{\mathrm{O}}_2}^{\mathrm{cal}}\right) $$

where k_L_a [h^-1^] is the volumetric oxygen mass transfer coefficient, L_O2_ [mol/L/bar] is the oxygen solubility, pO_2_^gas^ [bar] is the oxygen partial pressure in the headspace of the MTP and pO_2_^cal^ [bar] is the oxygen partial pressure used for calibration of the DOT measurement system (0.21 bar).

For the present calculations an equal OTR_max_ in MTPs and shake flasks is assumed for the compared conditions. Due to the intense ventilation of the culture chamber in the BioLector with air and the very high permeability of the applied sterile barriers (adhesive gas-permeable seal foils) [79] pO_2_^gas^ in the headspace of the MTP is assumed to be constant (0.21 bar) throughout the whole cultivation. The k_L_a values for the MTP were approximated by the smallest sum of squared errors (method of least squares) between the above described measured OTR vector from shake flask and the DOT-based OTR_calc_ values (Eq. 2) from MTP and are summarized in Table 2.

**Table 2:** Overview of the fitted k_L_a values

Cultivation	k_L_a [h^−1^]	Cultivation temperature [°C]	Shaking parameters			
			MTP^1^		Shake flask^2^	
			n [rpm]	V_L_ [mL]	n [rpm]	V_L_ [mL]
*E.coli* non-oxygen-limited	603	37	350	8	1500	1
*E.coli* oxygen-limited	183	37	350	25	800	1
*E.coli* diauxic growth	276	30	350	12	1000	0.6
*E.coli* induction	587	37	350	10	1500	1
*G. oxydans* unbuffered	425	30	350	10	1250	1
*G. oxydans* buffered	413	30	350	10	1250	1
*K. lactis* oxygen-limited	203	30	350	20	800	1

^1^all culltivations performed at a shaking diameter of 3 mm

^2^all culltivations performed at a shaking diameter of 50 mm

### Mass balance study

Calculation of carbon balances have been performed applying Equation 3

3 $$ {{\mathrm{q}}_{\mathrm{C}}}^{\mathrm{sub},\mathrm{i}} = {{\mathrm{q}}_{\mathrm{C}}}^{\mathrm{sub},\mathrm{r}} + {{\mathrm{q}}_{\mathrm{C}}}^{\mathrm{C}\mathrm{DW}} + {{\mathrm{q}}_{\mathrm{C}}}^{\mathrm{C}\mathrm{O}2} + {{\mathrm{q}}_{\mathrm{C}}}^{\mathrm{P}} $$

where q_C_^sub,i^ is the molar concentration of carbon in the initial substrate, q_C_^sub,r^ of the residual substrate, q_C_^CDW^ of the cell dry weight, q_C_^P^ of the product and q_C_^CO2^ the calculated integral of the CTR. For *E.coli* and *K. lactis* a biomass composition of C_1_ H_1.8_ O_0.5_ N_0.16_ and C_1_ H_1.85_ O_0.574_ N_0.22_ was assumed, respectively [80, 81].

### Offline analysis

The dell dry weight (CDW) was determined gravimetrically. The OD_600_ of the cell broth was measured in a Genesys 20 photometer (Thermo Scientific, Dreieich, Germany).

HPLC analyses were performed using the UltiMate 3000 from Dionex, Germany. The metabolites were separated on an organic acid resin column (250 mm × 8 mm) from CS-Chromatography Service (Langerwehe, Germany) at 60 °C. 5 mM H_2_SO_4_ was used as eluent at a flow rate of 0.8 mL/min. A refractive index detector Shodex RI-101 (Shodwa Denko Europe, Germany) was used to detect and record the peaks.

For the samples from *G. oxydans* the same device and column was used but the analysis was performed at 80 °C using 1 mM H_2_SO_4_ as eluent at a flow rate of 0.8 mL min^-1^. Glucose concentrations in these samples were quantified via an enzymatic assay (Boehringer Mannheim, Germany). To adapt the assay procedure to a 96 well microtiter plate, only 10 % of the specified volumes was used, reaching a target volume of 300 μL. The Synergy4 plate reader (BioTek Instruments, Bad Friedrichshall, Germany) was used to measure the absorbance at 340 nm.

## Additional files

Additional file 1: Figure S1. Determination of OTR_max_ for *E. coli* BL21 EcFbFP in shake flasks. Cultivation in WilmsMOPS mineral medium supplemented with 20 g/L glucose, buffered with 0.2 M MOPS in a 250 mL RAMOS shake flasks, V_L_ = 8–25 mL, n = 350 rpm, d_0_ = 50 mm, T = 37 °C. All measurements were performed in duplicates. Additional file 2: Figure S2. Determination of OTR_max_ for *E. coli* BL21 EcFbFP in MTPs. Culture conditions for microRAMOS: WilmsMOPS mineral medium supplemented with 20 g/L glucose, buffered with 0.2 M MOPS, 48-well Flowerplates, V_L_ = 0.6 - 1 mL, n = 500–1000 rpm, d_0_ = 3 mm, T = 37 °C. Arrows indicate the point at which the shaking frequency is shifted to the value written above. Additional file 3: Figure S3. Determination of OTR_max_ for *E. coli* BL21 EcFbFP in shake flasks. Cultivation in WilmsMOPS mineral medium supplemented with 10 g/L glucose, buffered with 0.2 M MOPS in a 250 mL RAMOS shake flasks, V_L_ = 8–25 mL, n = 350 rpm, d_0_ = 50 mm, T = 37 °C. All measurements were performed in duplicates.

## Abbreviations

2-KGA: 2-ketogluconic acid
5-KGA: 5-ketogluconic acid
a.u.: Arbitrary units
CTR: Carbon dioxide transfer rate
DOT: Dissolved oxygen tension
EcFbFP: *E. coli* modified FbFP
FbFP: FMN-based fluorescence protein
FMN: Flavin mononucleotide
k_L_a: Volumetric mass transfer coefficient
L_O2_: Oxygen solubility in the medium
MOPS: 3-morpholinopropane-1-sulfonic acid
MTP: Microtiter plate
OTR: Oxygen transfer rate
OTR_max_: Maximum oxygen transfer capacity
pO_2_^cal^: Oxygen partial pressure used for calibration
pO_2_^gas^: Oxygen partial pressure in the headspace of the MTP
RAMOS: Respiration activity monitoring system
RQ: Respiration quotient
S.L.: Scattered light
