# Supplementary material for: Parallel use of shake flask and microtiter plate online measuring devices (RAMOS and BioLector) reduces the number of experiments in laboratory-scale stirred tank bioreactors
Source: J Biol Eng. 2015 May 30;9:9. doi: 10.1186/s13036-015-0005-0 (PMC4531433; doi:10.1186/s13036-015-0005-0)
Supplement: Supplementary file 1 [file 13036_2015_5_MOESM1_ESM.docx]

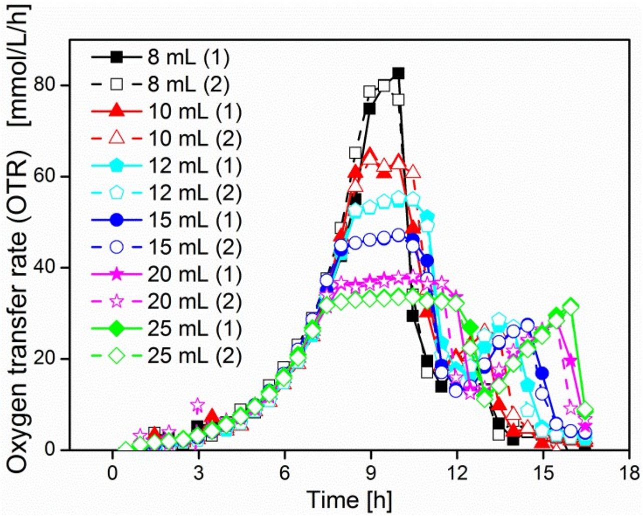

8 mL (1)
8 mL (2)
10 mL (1)
10 mL (2)
12 mL (1)
12 mL (2)
15 mL (1)
15 mL (2)
20 mL (1)
20 mL (2)
25 mL (1)
25 mL (2)
Oxygen transfer rate (OTR) [mmol/L/h]
0
20
40
60
80
0
3
6
9
12
15
18
Time [h]
