# Supplementary material for: Parallel use of shake flask and microtiter plate online measuring devices (RAMOS and BioLector) reduces the number of experiments in laboratory-scale stirred tank bioreactors
Source: J Biol Eng. 2015 May 30;9:9. doi: 10.1186/s13036-015-0005-0 (PMC4531433; doi:10.1186/s13036-015-0005-0)
Supplement: Supplementary file 2 [file 13036_2015_5_MOESM2_ESM.docx]

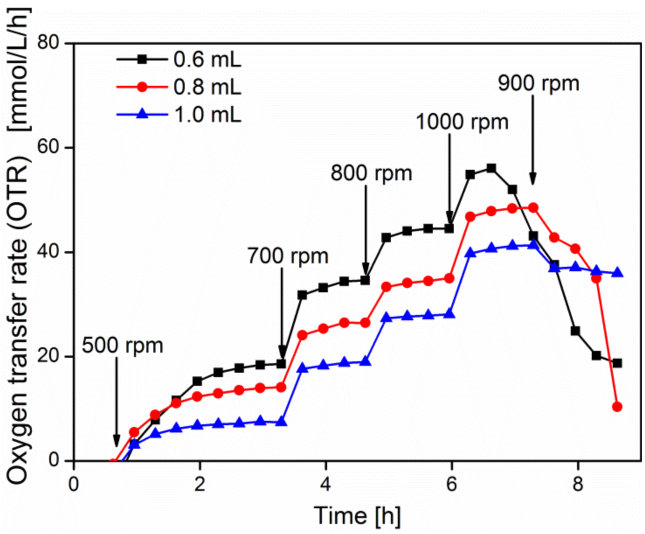

Oxygen transfer rate (OTR) [mmol/L/h]
Time [h]
0.6 mL
0.8 mL
1.0 mL
500 rpm
700 rpm
800 rpm
1000 rpm
900 rpm
0
20
40
60
80
0
2
4
6
8
